# Spectrum of HLA associations: the case of medically refractory pediatric acute lymphoblastic leukemia

**DOI:** 10.1007/s00251-012-0605-5

**Published:** 2012-02-15

**Authors:** William Klitz, Loren Gragert, Elizabeth Trachtenberg

**Affiliations:** 1 School of Public Health, University of California, Berkeley, CA USA; 2 National Marrow Donor Program, Minneapolis, MN USA; 3 Children’s Hospital Oakland Research Institute, Oakland, CA USA

**Keywords:** HLA, Pediatric, Acute lymphoblastic leukemia, Immunodominance, Allelic polymorphism, Mode of inheritance

## Abstract

Although studies of HLA and disease now date back some 50 years, a principled understanding of that relationship has been slow to emerge. Here, we examine the associations of three HLA loci with medically refractory pediatric acute lymphoblastic leukemia (pALL) patients in a case–control study involving 2,438 cases and 41,750 controls. An analysis of alleles from the class I loci, HLA-A and HLA-B, and the class II locus DRB1 illuminates a spectrum of extremely significant allelic associations conferring both predisposition and protection. Genotypes constructed from predisposing, protective, and neutral allelic categories point to an additive mode of disease causation. For all three loci, genotypes homozygous for predisposing alleles are at highest disease risk while the favorable effect of homozygous protective genotypes is less striking. Analysis of A–B and B–DRB1 haplotypes reveals locus-specific differences in disease effects, while that all three loci influence pALL; the influence of HLA-B is greater than that of HLA-A, and the predisposing effect of DRB1 exceeds that of HLA-B. We propose that the continuum in disease susceptibility suggests a system in which many alleles take part in disease predisposition based on differences in binding affinity to one or a few peptides of exogenous origin. This work provides evidence that an immune response mediated by alleles from several HLA loci plays a critical role in the pathogenesis of pALL, adding to the numerous studies pointing to a role for an infectious origin in pALL.

**Electronic supplementary material:**

The online version of this article (doi:10.1007/s00251-012-0605-5) contains supplementary material, which is available to authorized users.

## Introduction

At least as far back as the discovery and original interpretation of the simple beta-hemoglobin variant responsible for sickle cell disease (Allison 1954) the study of genetic variation and disease has been dominated by the concept that there is a best version of the alleles present at a single locus. “Wild type” is employed to refer to that optimal allele—self-defined by its high frequency in the population—with all other variation relegated to a “mutants” basket, or more recently “minor allele” category. While neutral variants and transitional and balanced polymorphisms are now recognized as contributors to empirically recorded genetic variation, the dichotomy of good genes and bad genes remains.

The HLA complex of the major histocompatibility complex of vertebrates with its multiple linked and highly polymorphic loci may challenge that conception. Interestingly, HLA studies (now followed through the tens of thousands of publications on HLA and disease) have been dominated by the recording—allele by allele—of individual single gene–disease relationships. Yet a strength of the HLA complex for genetic studies is that a great deal is now understood of HLA function down to the level of individual alleles. Each HLA allele can be characterized in terms of its peptide binding capacity for initiating the immune response and in the case of alleles of the HLA class I loci, its ligand status for alleles of the unlinked natural killer cell immunoglobulin-like receptor (KIR) system (Bashirova et al. 2011). Taking advantage of this background context, we evaluate the relationship of HLA variation to disease susceptibility in the predominant pediatric oncologic disease. Our particular emphasis is on the population level consideration of the contributions of all HLA alleles present.

The first disease shown to have an association with the major histocompatibility complex (MHC or HLA in humans) was in murine models with leukemia (Lilly et al. 1964). The first human disease found to be associated with HLA was childhood acute lymphoblastic leukemia (ALL) (Walford et al. 1970); HLA serogroups A2 and B12 were also found in a cancer family syndrome (Lynch et al. 1975). Further evidence for the association of HLA with leukemia came from atypical HLA segregation patterns observed in leukemic families which show: (1) an increase in HLA-identical non-affected sibs, (2) HLA homozygosity, and (3) maternal class II DRB1 identity of disease association haplotypes (Ryder et al. 1979; Sendid et al. 1998; reviewed in Taylor et al. 2009). The lack of a disease-specific segregation in families, however, suggests that HLA is not directly responsible for leukogenesis but may play a role in susceptibility to development of leukemia or may be a marker in linkage disequilibrium with another disease gene. Not all studies of leukemia and HLA demonstrate associations, however (Hosking et al. 2011).

The evolution of infectious agents and their human hosts has been a dynamic process. The maintenance of the extreme polymorphism of the HLA has played an important role in increasing the opportunity of survival when combating an ever-changing variety of pathogenic organisms. HLA may contribute to the risk of developing leukemia not only by recognition and presentation of viral or other peptides to cytotoxic T cell lymphocytes and NK cells but also by escape from host immune surveillance mechanisms (e.g., HLA class I downregulation associated with breast and ovarian cancer (Vitale et al. 2005, 1998)), and it may reflect accumulation of abnormalities associated with disease progression. HLA genotypes may play a significant role in the modification of environmental risk and thereby influence the development of high risk pediatric ALL.

The most common form of cancer in children is acute lymphocytic leukemia, which comprises over 70% of cancers in the pediatric population worldwide (Goldberg et al. 2003). Analysis of HLA in relatives or in unrelated stem cell donor registries is often requested to determine if an appropriate immunogenic match exists for ALL patients who are not responding well to therapy and thus at high risk. We examined European American ALL patients ages 2–16 from records of patients being evaluated for possible stem cell transplantation, designated pediatric acute lymphoblastic leukemia (pALL). It is estimated that the great majority of these pALL patients have precursor B cell ALL, as precursor T cell ALL is much more difficult to reach second remission once relapsed, as required for transplantation. Infants (<2 years) with ALL were not examined, although they are the group with the highest risk of treatment failure because they have distinctive types of precursor B cell and mixed lineage phenotypes with a different, predominantly genotoxic phenotype (Greaves 1996; Greaves and Wiemels 2003). Utilizing data from medically refractory pALL patients and adult controls matched for ethnicity, sex, and geographic origin from the National Marrow Donor Program (NMDP), we studied the influence of variation at the HLA class I loci (HLA-A and HLA-B) and the HLA class II DRB1 locus on susceptibility to this common malignancy of childhood.

By testing and comparing the pALL susceptibilities of each allele at each of three HLA loci in the population as a whole, we uncover a near continuous array of protective and predisposing allelic effects influencing disease outcome at each locus. Based on studies of carefully examined diseases with known immunogenic peptides differentially bound by HLA molecules, our evidence suggests that development of pALL is associated with a population level response of HLA to infectious disease. The results of HLA association with pALL fit well with a body of work suggesting an infectious origin of this childhood cancer.

## Results

### Allele effects by locus

The influence of any HLA allele on pALL may be protective, predisposing, or neutral (exerting no effect). The impact of HLA on medically refractory pALL is examined here by analysis of individual allelic influences for each class I HLA-A, HLA-B, and class II DRB1 loci, typed at two-digit resolution in a Caucasian sample of 2,438 high-risk pALL patients (4,876 chromosomes) and 41,750 controls (83,500 chromosomes). The relationship of each allele to pALL is expressed through an odds ratio (OR) calculated in the context of all alleles at each locus. The impact of each locus is revealed through the overall significance of the deviation of the table consisting of the k alleles for a locus by case and control frequencies. To help prevent the inclusion of possibly spurious influences due to small sample sizes, for inclusion as an individual category an allele must have at least 50 observations in cases and controls combined. The results of this test on each of the three loci are shown in Table 1. For the HLA-A locus, the median sample size of the 20 HLA-A alleles is 2,317. The overall log likelihood ratio or G test value is 145.3 which, with 19 degrees of freedom, results in a *p* value of 1.8E-21. Three rare alleles of HLA-A (A*36, A*43, and A*80) were excluded. For HLA-B, the single allele test had 27 non-rare alleles, a median size of 1,749, and a *p* value of 1.4E-55, with nine HLA-B alleles too rare for individual inclusion. For the class II DRB1 locus, all 12 alleles qualified for testing with a median category size of 9,899 and a *p* value of 1.8E-28. Each of the tests indicates extremely significant differences in the distribution of patient and control allele frequencies.

**Table 1:** Pediatric ALL cases versus controls: summary of overall significance testing

Genetic factor	pALL cases	Controls	Median no., samples per category	*k*	G	*P* value	Total no. favorable, predisposing, and neutral groups
Alleles	2n	2n					
HLA-A	4,876	83,500	2,317	20	145.3	1.8E-21	
HLA-B	4,876	83,500	1,749	27	335.7	1.4E-55	
DRB1	4,876	83,500	9,899	12	159.4	1.8E-28	
Genotypes	Number	Number					
HLA-A/HLA-A	2,363	40,710	193	95	221.6	2.2E-29	6
HLA-B/HLA-B	2,169	38,410	147	162	403.2	9.3E-23	6
DRB1/DRB1	2,404	41,485	369	65	192.7	8.0E-15	6
Haplotypes	2n	2n					
HLA-A~HLA-B	4,876	83,500	183	200	798.2	1.1E-72	8
HLA-B~DRB1	4,876	83,500	404	180	1096.1	1.6E-131	8

Rare category alleles (i.e., <50 observations) include HLA-A *36, *43, *80; HLA-B *46, *54, *59, *67, *73, *78, *81, *82, *83; DRB1 none. *k* is the number of rows (genetic category) in 2 × *k* test of heterogeneity using the log likelihood or G statistic. The degrees of freedom for this test are (*k* − 1). Genotype and haplotype categories assembled from significance of HLA-A, HLA-B, and DRB1 allelic testing are categorized as favorable–protective (*f*), predisposing (*p*), or neutral (*x*)

**Fig. 1:**
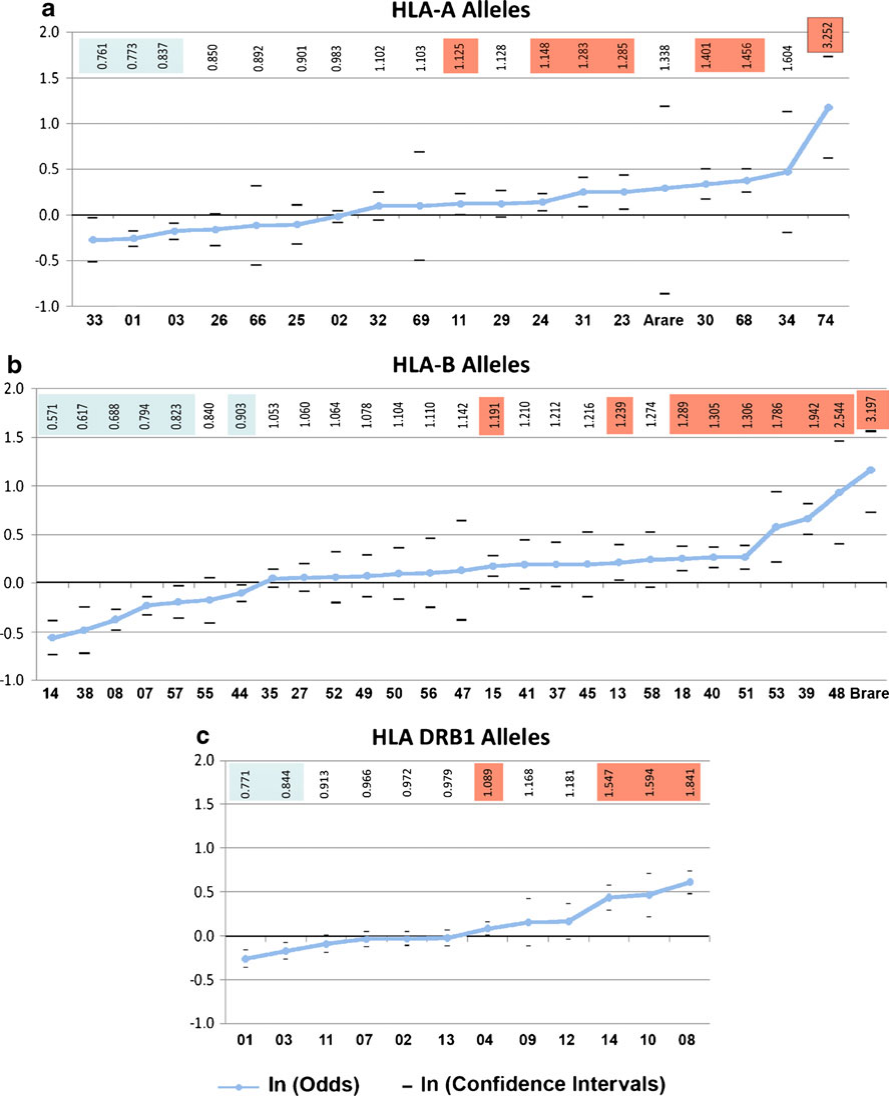
A continuum of pALL risk by HLA alleles at HLA-A, HLA-B, and DRB1. Extreme statistical deviation between cases and controls is present at each of the three loci (see Table 1 and Tables S2–S4) with at least half of the alleles at each locus are either significantly protective (*blue*) or significantly predisposing (*orange*). Odds ratio values are listed at the top of each allele, with the vertical scale plotted in ln(Odds) to allow positive and negative odds representation on the same scale

Association analysis of HLA-A reveals the 20 sampled alleles ranging from odds ratios of 0.761 to 3,252 (Fig. 1a). Based on an individual significance criterion of *p* < 0.01, four HLA-A alleles (A*33, A*01, A*03, and A*26) are significantly protective, and six alleles predispose to high-risk pALL (A*24, A*31, A*23, A*30, A*68, and A*74). Overall, the 20 alleles at HLA-A indicate a continuum of relationships, with the sense that larger sample sizes might clarify the predisposing or protective status of several alleles whose confidence intervals are close to, or include, an odds ratio of 1.0. For the HLA-A locus, 50% of alleles as a whole are either significantly predisposing or protective.

HLA-B with its intrinsically greater allelic polymorphism has a total of six alleles significantly protective (B*14, B*38, B*08, B*07, B*57, and B*44) and eight predisposing to pALL (B*15, B*18, B*40, B*51, B*53, B*39, B*48, and the assembled rare allele category “B*rare”). In the diagram of HLA-B alleles sorted by odds ratios (Fig. 1b), we again see a continuum of relationships to high-risk pALL susceptibility with odds ratios ranging from 0.571 to 3.197, and 76% of alleles significantly deviant from OR = 1. As was true for HLA-A, it appears that, taken as a whole, predisposing HLA-B alleles outnumber those conferring protection. For the HLA-B locus 24.0% of alleles are neutral with respect to disease.

Analysis of the HLA class II DRB1 locus reveals 12 alleles (Fig. 1c) in a similar continuous range, except that the predisposing alleles DRB1*14, DRB1*10, and DRB1*08 depart more dramatically from the continuous change in susceptibility seen in the other nine DRB1 alleles. In addition, the common and heterogeneous allele DRB1*04 is also slightly predisposing (OR = 1.089). For HLA class II DRB1, six of the 12 alleles are involved in disease protection or predisposition with odds ratios ranging from 0.771 to 1.841.

Identified effects from each allele must be provisionally regarded as marker effects, rather than directly contributing to the disease. The two-digit HLA typing resolution available in this study hides considerable and often demonstrably consequential additional genetic variability. We examine subtypes of the protective alleles HLA-B*44, known to be composed of many distinct alleles when examined at higher resolution. Two of these are especially common in European Americans (B*44:02 at 9% and B*44:03 at 5% frequency (Maiers et al. 2007)). Examining a large sample of high-resolution-typed three locus haplotypes (Maiers et al. 2007) shows that the two common haplotypes A*29~B*44~DRB1*07 and A*02~B*44~DRB1*04 effectively refer to the high-resolution versions of the two most common HLA-B*44 haplotypes; A*29:02~B*44:03~DRB1*07:01 has a population frequency of 0.019, while A*02:01~B*44:02~DRB1*04:01 has a frequency of 0.026. The suggestion that the HLA-B*44:02 haplotype may be the primary source of the protective HLA-B*44 signal may be tested by comparing the odds ratios of the HLA-B*44:02 and HLA-B*44:03 alleles identified through their three-locus haplotype proxies. The overall three locus haplotype pALL risk of A*29~B*44~DRB1*07 is non-significant at OR = 1.05 (confidence interval (CI) = 0.84, 1.32). In contrast, the A*02~B*44~DRB1*04 haplotype is significantly protective at OR = 0.67 (CI = 0.54, 0.83), with the confidence intervals of the two haplotypes non-overlapping. The protective effect of the HLA-B*44:02 allele can be interpreted as due to its carriage of a protective influence above and beyond the HLA-B*44 defined marker alone.

### Genotype effects

From the view of trait inheritance, an allele can act on its own and in combination with its allelic homologue. For HLA, these possible effects can also be extended to consideration of alleles at other loci in the same gene family, for example, the class I loci HLA-A and HLA-B. Allelic influence can also be a continuum of effect magnitudes from protective to neutral to predisposing. To better understand the influence of HLA on high-risk pALL, we start by analyzing these possible higher order interactions by focusing on the nominally significant predisposing and protective alleles identified from the single locus analyses of HLA-A, HLA-B, and DRB1.

Using the identified single allele influences [favorable–protective (f), neutral (x) and predisposing (p)] on pALL, we divide the possible HLA-A influence into a number of qualitative categories. For a genotype of HLA-A, HLA-B, or DRB1, an allele may act alone in the neutral heterozygotes (px or fx), as homozygotes (ff, pp, or xx), or as mixed heterozygotes (fp). For the haplotypes HLA-A−HLA-B and HLA-B−DRB1, similar categories can be defined, with the expansion of the two single allele effect heterozygotes to one for each of the loci making a total of eight possible categories. We thus tally case and control numbers for six genotypic and eight haplotypic categories and calculate the odds ratios for HLA association with pALL. The comparison of genotypic and haplotypic categories permit testing of interaction effects within and between loci, locus-specific differences in protection and causation and the nature of interaction when protective and predisposing alleles occur together, i.e., the mode of inheritance and interlocus effects.

Even with the 50-sample minimum size restriction on testable genetic entities, 65 to 162 distinct testable genotypes per locus are available, while the median number of samples per category drops from 369 to 147 (Table 1). The overall genotype case–control G tests are again extremely significant for all three loci.

**Fig. 2:**
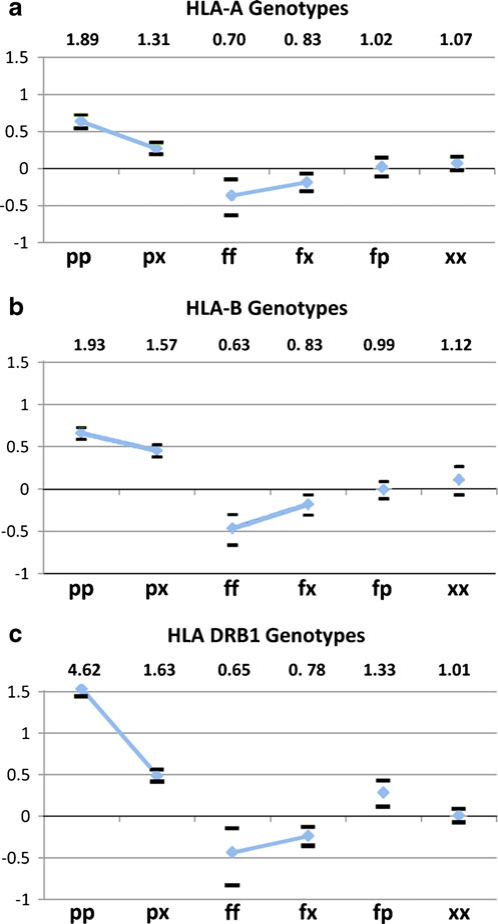
Mode of inheritance of pALL risk in HLA revealed by HLA genotypic groups defined by allelic predisposition. Genotypic groups are defined from the individual allele results for each locus (Fig. 1 and Tables S5–S7). Homozygous predisposing genotypes (*pp*) are at greater risk than heterozygous predisposing genotypes (*px*) at all three loci **(a–c)**. For DRB1 the pp genotype is especially predisposing. Homozygous favorable–protective genotypes (*ff*) always confer greater protection at each locus than the heterozygous fx genotypes but never significantly so. The mixed fp genotypes have OR = 0 for HLA = A and HLA-B, but for DRB1 fp OR > 1. The neutral homozygotes xx have OR = 0 at all three loci. For the mixed genotypes (fp) odds = 1 for HLA A and HLA B, while for DRB1 odds >1. Both predisposing and protective inheritance appear to be intermediate with predisposition stronger than protection. Odds ratio values are listed at the top, with the vertical scale plotted in ln(Odds) to allow positive and negative odds representation on the same scale. *p* predisposing allele, OR > 1; *f* protective–favorable allele, OR < 1; *x* neutral allele, OR = 1; *mixed* refers to genotypes bearing both a *p* and *f* allele

Genotypic groups are defined from the individual allele results for each locus (Fig. 1 and Supplemental Tables). These are classified according to allelic composition (p, predisposing allele: OR < 1; f, favorable–protective allele: OR > 1; x, neutral allele: OR = 1). Beginning with a comparison of the six HLA-A genotype categories (Fig. 2a), we see that single allele effects of protective-neutral heterozygotes (fx) (identified as alleles HLA-A*01 and HLA-A*03)) are significantly protective (OR = 0.83), as are the protective homozygotes ff (OR = 0.70). Because the protective and predisposing genotypes are made up of those significantly deviant alleles the direction of the deviations are expected; the odds of the protective HLA-A homozygotes are lower than the protective neural genotypes, but not significantly so.

Summarizing the genotypic outcomes from all three loci (Fig. 1a, b, c), we note that homozygous predisposing genotypes (pp) are at greater risk than heterozygous predisposing genotypes (px) at all three loci (Fig. 2a, b, c); as described in “Methods,” here we use a natural logarithm (ln) of the odds to allow positive and negative odds representation on the same scale. For DRB1 the pp genotype is especially predisposing. Homozygous favorable–protective genotypes (ff) always confer greater protection at each locus than the heterozygous fx genotypes, but never significantly so. The mixed fp genotypes have OR = 0 for HLA = A and HLA-B, but for DRB1 fp OR >1. The neutral homozygotes xx have OR = 0 at all three loci. For the mixed genotypes (fp) odds = 1 for HLA A and HLA B, while for DRB1 odds >1. Both predisposing and protective inheritance appear to be intermediate with predisposition stronger than protection.

### Haplotype effects

**Fig. 3:**
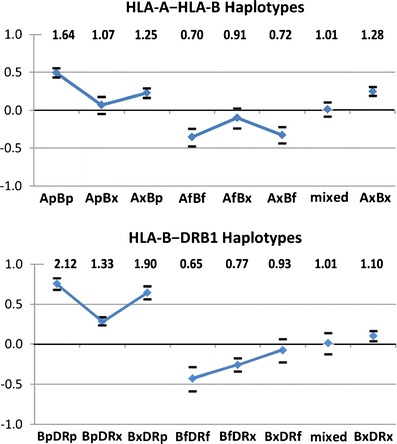
The pALL risks of two locus haplotype groups defined by allelic predisposition reveal locus-specific influences. Haplotype groups are defined from the individual allele results for each locus (see Fig. 1 and Tables S8 and S9). The six predisposing-allele bearing haplotypes pp, px, and xp of A~B and of B~DR all have OR > 1. Examining the predisposing haplotypes more closely shows that the two pp bearing haplotypes (Ap~Bp and Bp~DRp **(a, b)**) each have greater predisposition than the heterozygous px and xp haplotypes. The two Bx haplotypes predisposing due to linkage (Ap~Bx and Bx~DRp) have contrast levels of pALL predisposition at OR = 1.07 and 1.90, respectively, with Bx~DRp possessing much higher predisposition than Ap~Bx, while the two Bp-only haplotypes are very similar at OR = 1.25 and 1.33. Haplotypes bearing favorable or protective alleles reveal a different pattern. Of the three A–B and B–DRB1 protective haplotypes bearing favorable–protective alleles (f), only those bearing a protective HLA-B allele have OR significantly <1, with the Af~Bx and Bx~DRf haplotype risks not different from OR = 1. The *mixed* haplotypes (i.e., fp and pf of A–B and B–DRB1 **(a, b)**) are each very close to OR = 1, suggesting that predisposing and protective effects on the same haplotype cancel each other out. The two xx haplotypes are each protective, implying locus interactions not evident in the ORs of the single alleles from which they are derived. While all three loci appear to influence pALL, HLA-B influence is greater than HLA-A while DR predisposition is greater than HLA-B predisposition. Odds ratio values are listed at the top, with the vertical scale plotted in ln(Odds) to allow positive and negative odds representation on the same scale. *p* predisposing allele, OR > 1; *f* protective–favorable allele, OR < 1; *x* neutral allele, OR = 1; *mixed* refers to Bp~DRf and Bf~DRp haplotypes combined

Haplotype groups are defined from the individual allele results for each locus (see Fig. 1 and Supplemental Tables). As seen in Fig. 3, the six predisposing allele-bearing haplotypes pp, px, and xp of A~B and B~DR all have OR >1. Examining the predisposing haplotypes more closely shows that the two pp bearing haplotypes (Ap~Bp and Bp~DRp, Fig. 3a, b) each have greater predisposition than the heterozygous px and xp haplotypes. The two Bx haplotypes predisposing due to linkage (Ap~Bx and Bx~DRp) have contrasting levels of pALL predisposition (OR = 1.07 and 1.90, respectively), with Bx~DRp possessing much higher predisposition than Ap~Bx; this is in contrast to the two Bp predisposing haplotypes (Ax~Bp and Bp~DRx) that are very similar in risk (OR = 1.25 and 1.33).

Haplotypes bearing favorable–protective alleles reveal a different pattern. Of the three A~B and B~DRB1 protective haplotypes, only those bearing a protective B allele have an OR significantly less than 1. In contrast, the neutral Bx haplotypes (Af~Bx and Bx~DRf) are very similar in risk and not different from OR = 1. The mixed haplotypes (i.e., fp and pf of A~B and B~DRB1; Fig. 3a, b) are each very close to OR = 1, suggesting that predisposing and protective effects on the same haplotype cancel each other out. The two neutral xx haplotypes are each slightly protective, implying locus interactions not evident in the ORs of the single alleles from which they are derived. While all three loci appear to influence pALL, HLA-B influence is greater than HLA-A in both protective and predisposing directions, while at DRB1 the effect is strongly predisposing.

Taken as a whole, these results reveal several new characteristics of the action of the three HLA loci in medically refractory pALL. The influence of HLA-A alleles on pALL causation is seen to be weakest from both the genotypic and haplotypic evidence, although HLA-A predisposing genotypes do have a greater effect than predisposing-neutral genotypes, suggesting a possible true HLA-A locus signal in this instance. The HLA-A influence on pALL protection was relatively weak and disappeared entirely in the HLA-A–allele alone comparisons for HLA-A~HLA-B haplotype predisposition. HLA-B alleles demonstrated both substantial protective and predisposing effects with a consistent increase in effect due to homozygosity of protective and predisposing alleles. Viewed against the background of B~DRB1 haplotypes, HLA-B alleles maintained evidence of both protection and predisposition. For DRB1 genotypes both protection and predisposition was present, but only the doubly predisposing genotypes were of greater influence than those having a single predisposing allele. From the B~DRB1 haplotypes, DRB1 protection appears weak, but the predisposing DRB1 alone and B~DRB1 genotype effects were very strong and did not differ. This suggests the possibility that the class II signal may be primarily or even exclusively predisposing to disease.

## Discussion

We have examined a large sample of medically refractory pediatric ALL patients and compared them to a set of carefully constructed controls at two HLA class I loci (HLA-A and HLA-B) and one HLA class II locus (DRB1). For each locus, the alleles present in the population show a continuous range of associations with high-risk pALL, from strongly predisposing to strongly protective (Fig. 1). Many of the alleles at each locus were found to be individually deviant from a neutral effect. Knowledge of differential binding and presentation of peptide antigens to the immune system to trigger T cell-mediated immune responses helps clarify a population level understanding of HLA alleles in disease predisposition. A single pathogen-derived peptide associates with the HLA alleles at a locus across a spectrum of binding affinities for the exposed individuals in a population. This results in variation in disease outcomes, depending in part on the HLA/peptide characteristics accruing from each individual’s HLA composition.

An immune response characterized by domination of just one or two peptides of the many hundreds present in an invading pathogen—termed immunodominance—is the most common pattern encountered for both CD8+ (HLA class I) and CD4+ (HLA class II) immuno-responses (Richeldi et al. 1997; Yewdell and Bennink 1999). We infer that the pattern of population-wide allelic association represented here may well reflect the effects of immunodominance; if the effectiveness of several HLA alleles differed for a number of similarly reactive peptides, then a clearly recognizable range of associations would not be evident. While several steps in normal antigen presentation, ranging from antigen processing to the survival of peptide MHC attachment on a lymphocyte on the way from the periphery to a lymph node can influence immunodominance, the key factor is the kinetics and stability of the peptide/MHC bond itself (Lazarski et al. 2005; Rosenman et al. 2011; Van Dyke et al. 2011; Weaver and Sant 2009).

The spectrum of binding affinities for HLA-A and HLA-B in relation to viral proteins in the context of host–pathogen co-evolution shows a general tendency for high binding efficiencies to be associated with conserved proteins (Hertz et al. 2011). This work also illustrated that HLA alleles susceptible to dengue virus also had the lowest binding affinities, and similarly, rapid progression of HIV-1 was associated with HLA alleles having low binding affinity to the gag protein. Another pertinent aspect of this work is that HLA-A and HLA-B loci were shown to be capable of distinct peptide binding relationships to a given virus. This suggestion of an evolutionary division of labor between the two loci is echoed in the finding here of differential influences of HLA-A and HLA-B, as well as DRB1 (Figs. 2 and 3).

Studies on influenza virus immunity have been valuable in elucidating the MHC/peptide relationship and provide insights on the intricacies of immunodominance. Across the five protein sequences of influenza virus, immunodominance due to peptides derived from two proteins was observed to occur in two distinct class II MHC molecules (Nayak et al. 2010; Snyder et al. 2008). Even with modest overlap in CD4 + -specific reactivity between the two MHC molecules, a pattern of high to low binding affinities across a population’s allelic repertoire could produce two (or more) highly protective alleles with graded degrees of protection as seen in our present study.

A case study of class II HLA variation and a simplified immunogenic stimulus resulting in disease can be found in chronic beryllium disease, a T cell-mediated lung condition caused by hypersensitivity to beryllium due to genetic variation at HLA class II DPB1 after beryllium exposure. Only those individuals bearing glutamic acid at DPB1 position 69 of the antigen binding pocket P4 are susceptible (Richeldi et al. 1997). But, reflecting the subtleties typically present in HLA diseases, this relationship is influenced by additional DRB1 and DPB1 genetic differences and environmental variation (Rosenman et al. 2011; Van Dyke et al. 2011). The P4 antigen-binding pocket of the DP molecule with those DPB1 alleles having highest negative regional surface charge creates highest susceptibility to chronic beryllium disease followed by alleles with a relatively lower charge valence, while a set of alleles with the lowest valence contribute negligibly to susceptibility (Snyder et al. 2008). Thus, the binding strength of the beryllium atom in the P4 pocket varies as a function of the local charge of the DP molecule. Environmental factors (duration of beryllium exposure and smoking) have the effect of smoothing the differences in susceptibility among DPB1 genotypes. Thus, this example demonstrates the action of a single antigenic factor leading to a range of HLA predispositions underlain by differential antigen binding among HLA alleles.

A study directly pertinent to HLA–pathogen has been clearly shown in research on the *Borrelia burgdorferi* immunodominant OSPA peptide to DRB1 alleles in a sample of individuals divided into patients responsive and refractory to antibiotic treatment of Lyme arthritis (Steere et al. 2006). Ordering patients by the experimentally determined OSPA binding strength of the most common 14 DRB1 alleles in the European American population under study revealed a continuous range of outcomes with the binary responses to antibiotic treatment (responsive or refractory) correlated with the binding affinity of the DRB1 alleles they carried. More complex patterns of peptide binding and stimulation of T cells created by a plurality of pathogenic peptides would tend to mask such relationships, as different peptides will likely bind most efficiently to different alleles.

If a range of binding affinities among HLA alleles to a peptide, as discussed here, is the norm, then the demonstration of a spectrum of differences in a case–control study will require sufficient sample sizes to detect not just one most deviant allele positively associated with disease but the documentation of a full range of associations, including negative associations that are statistically more difficult to detect. Thus, studies employing smaller sample sizes will only be capable of the identification of just one or a few of the most common and most strongly positively disease-associated alleles, as is the case for the great proportion of HLA–disease studies over the last decades.

### Infectious etiology

Our work reveals the action of a strong immune response mediating the outcome pathogenesis of high-risk pALL and complements a number of studies carried out over the last several decades on the role of infectious agents in pediatric leukemias. The body of epidemiologic evidence indicates that the two major subtypes of pALL (precursor B and T cell) have different etiologies (Davies et al. 2004; Eden 2010; Goldberg et al. 2003; Greaves 2006, 1997; Uckun et al. 1998). These leukemias are likely a result of both environmental and genetic factors, with the risk of developing leukemia involving multiple genes conferring susceptibility to the development of malignancy. There is now strong evidence that immunologic mechanisms may be involved in the etiology of malignancy in pediatric precursor B cell ALL and therefore a comprehensive evaluation of the association of the HLA system with disease susceptibility is of considerable interest. The HLA system interacts with infectious and environmental agents and presents foreign peptides to circulating cytotoxic T lymphocytes and natural killer (NK) cells. It is the HLA + peptide + T cell receptor restriction complex that acts to determine “self” and “non-self” antigens, and modulates the resulting immune response. HLA-influenced modulation of immune response may play a critical role in the development of B cell pALL through differential antigen presenting capabilities and ensuing immunologic reactions.

Epidemiologic factors from lifestyle indicators (including socioeconomic status, parental occupation, urban versus rural status), disease clustering as in Kinlen’s “population mixing” hypothesis (Kinlen 1988, 1997), and the timing of exposure to infection, as in Greave’s “delayed-infection” hypothesis (Greaves 1993, 1988, 1997) all support a role for infection in the etiology of pALL. In common pediatric ALL, a two-step susceptibility model is often used to describe its etiology, with the first step including an in utero chromosomal rearrangement followed by a second postnatal “hit.” In this hypothesis, genetic susceptibility, which by itself is not uncommon, is neither sufficient nor necessary but may contribute to the risk, acting as a promoter rather than an initiator of neoplastic disease (Taylor et al. 2008; Greaves 2006). Greaves and others also hypothesized that early life exposure to infection is essential to organize the adaptive immune complex into an efficient response system; a delay in exposure to infection could thus result in proliferative stress on pre-leukemic stem cells leading to further mutations and malignancy (Greaves 2006, 1997; Greaves and Alexander 1993; Taylor 1994; Thorsby 1997). This theory is in accord with the “hygiene hypothesis” and immunologic organization, whereby the lack of early infections results in a disorganized immunologic system with resultant autoimmune disease. There is also a correlation between childhood allergies, type I diabetes, and pALL which may mean that they share common infectious risk factors. A substantial body of epidemiologic data exists supporting an infectious etiology of pALL (Greaves 2006; Little 1999; McNally and Eden 2004; O'Connor and Boneva 2007). Moreover, recent evidence on exposure to common childhood infections, as measured by day care attendance, birth order, and common childhood infections in infancy strongly supports a protective role for early infection in common childhood ALL (Urayama et al. 2010).

### Interpreting HLA genetics

We have shown that, even at the relatively stringent cutoff of *p* < 0.01, at least half of the alleles at each of HLA-A, HLA-B, and DRB1 are significantly associated with either predisposition to or protection from medically refractory pALL. A pattern of a continuum of risk association to the disease is present for alleles at each of the loci. Taken as a whole, the distributions of frequencies of alleles at each of the loci in patients and controls are shown to be extremely different using formal statistical tests. Genotypic groupings—categories of genotypes based on the identified deviant alleles−demonstrate that homozygous effects (revealing additive or intermediate modes of inheritance) are most striking in the predisposing alleles of each locus. Attributions of interlocus effects among loci are explored with disease risks of the two locus haplotypes A~B and B~DRB1. The HLA-A allelic contribution in the haplotype context is less than that of HLA-B alleles for both protective and predisposing effects, while at the same time predisposing alleles from both HLA-A and HLA-B loci on the same haplotype have a significantly greater effect than HLA-A or HLA-B alleles alone. For B~DRB1 haplotypes, the B~DRB1 combination of two protective alleles is significantly more protective than that of a haplotype having only one HLA-B or one DRB1 predisposing allele. This suggests that HLA-B and DRB1 alleles may synergistically increase protection. For the predisposing B~DRB1 haplotypes, DRB1 effects were shown to greatly exceed those of HLA-B-only haplotypes. This evidence points to a distinct action of the class II DRB1 locus variation in disease susceptibility relative to that of class I loci.

Although the haplotype analysis here suggests contribution from linked loci in the development of pALL, there are additional tightly linked polymorphic candidate HLA loci that may be contributing to the observed effects. Notable HLA class I and class II loci in high linkage disequilibrium and tight linkage with two of the loci are examined here: HLA-C with HLA-B and the DQA1~DQB1 loci with DRB1. Variation at all three of the HLA class I loci (HLA-A, HLA-C, and HLA-B) play a role in determining the activity of the NK cell system of KIR loci, and this is especially true of HLA-C. Some of the HLA-A and HLA-B influences on pALL may be indirect influences emanating from the unlinked KIR system acting through the class I loci (Bashirova et al. 2011). Finally, we mention the possibility of indirect influence, again through linkage disequilibrium, of non-HLA immune function loci within the HLA complex, including tumor necrosis factor alpha (TNF-α).

In the current study, we have identified a continuum of susceptibilities in alleles of three HLA loci with pALL. We argue that one explanation for this pattern is variable pathogen binding under a regimen of immunodominance. Various avenues of future work can be suggested. It would be good to confirm the basic HLA-A, HLA-B, and DRB1 results with coincident studies on the other class II loci and HLA-C, as well as simultaneous genotyping of KIR. HLA-C and DQ loci are in strong linkage disequilibrium with HLA-B and DRB1, respectively, so a first order evaluation of the roles of these loci should be possible. Stem cell registries, because of their large sample sizes and quality controls, are a good source of material for studies on additional transplantable diseases that could readily take place under this framework. Also, in this regard, the hypothesis of variable binding of a single epitope of pathogenic origin could be examined with bioinformatics tools comparing the range of allelic associations at a locus with known binding characteristics of each allele. Finally, efforts to uncover the responsible infectious agent(s) underlying pALL must be renewed.

## Methods

### Case and control samples

The two major subtypes of pediatric ALL are defined by different cell lineages. The precursor B cell lineage predominates at >85% of pALL with a frequency peak between the ages of 2 and 5. The much rarer precursor T cell form is found in ~10–15% of pALL and more frequently in older children of non-European heritage (Uckun et al. 1998). Treatment regimens for the characteristically more severe precursor T cell pALL have improved over the years so that it is no longer an adverse risk factor (Davies et al. 2004; Goldberg et al. 2003; Uckun et al. 1998). While most of the pediatric ALL cases are cured by chemotherapy, approximately 10–15% of the cases are refractory to that treatment and are then often referred for transplantation with hematopoietic stem cells. Within the group of pALL patients that are medically refractory and considered for stem cell transplantation, the precursor B cell versus precursor T cell lineages mirror the frequency of these types in the pALL population as a whole. It is often difficult to get T cell lineage pALL patients into remission after relapse, as required for transplantation, leaving the majority of the patients advancing to stem cell transplantation with pre-B cell leukemia.

Pediatric ALL cases in this study consist of all European American patients for whom preliminary hematopoietic stem cell transplantation donor searches of the National Marrow Donor Program registry was performed with ALL stated as the disease. In that most pediatric ALL is cured by chemotherapy, the present analysis is skewed toward more severe disease with the potential for hematopoietic stem cell transplantation. Patients were chosen within the age range of 2–16 years which would capture the most typical pALL cases. HLA data from the donor controls and cases were performed using consistent DNA methods between the years of 1997 and 2002. Table S1 defines the sample numbers, male/female frequencies, and breakdown according to geographic region tested.

Controls include sample data from adult donors (18–29 years) recruited by the NMDP who self-identified as European American (Table S1). Improperly matched control datasets in HLA disease association studies can create erroneous correlations that are simply artifacts of differential geography, gender, age, birth date, and ancestry between cases and controls. Geographical variation in HLA across the USA has been well documented, so the geographical distribution of the control population should match the distribution of the patients. Similarly, while HLA and gender effects are very minor, they are considerably magnified by the combination of the preponderance of females in the donor pool and the increase of males in the pALL patient cohort. Differences in age and birth date exist because of differing patterns of immigration and admixture within self-identified groups over time as well as protective/predisposing HLA-related effects on human survival. Finally, there can be an underlying population substructure beneath the level of self-identified race. In our initial analyses, we documented an excess of Jewish ancestry in the European-American patient population that was corrected by removing individuals with likely Jewish ancestry from both the case and control sets.

Based on Ashkenazi haplotype frequencies from the Hadassah registry (Klitz et al. 2010), the European-American patient sample was calculated to be 10% Jewish, compared to 5% Jewish for the control sample. Jewish haplotypes were then sampled from the Ashkenazi frequencies with replacement and randomly selected subjects with the haplotype were removed from both the patient and control samples until the estimated Ashkenazi admixture was 0% in both patient and control samples. This brought the patient sample from 2,868 to 2,438 individuals and the control population from 433,838 to 395,838 individuals.

The USA is split into four geographical regions based on the first digit of the zip code (0,1 = east; 2, 3, 7 = south; 4, 5, 6 = midwest; 8, 9 = west). Controls were subtracted optimally until the distribution of controls in each region matched the patient distribution. For the final step, after the geographical matching of controls, females were removed from the control sample until the control gender ratio matched the patient gender ratio. For gender, the ratio of males to females in the patient sample was 1.59. To control for age, the controls over age 30 were removed from the sample, leaving only controls between age 18 and 29. Of 393,838 European-American controls, 341,291 remained after geographical matching, 144, 142 remained after gender matching, and 41,750 after controlling for age (Table S1).

### Statistics

The overall difference between an HLA genetic category (alleles at a locus, genotypes, or two-locus haplotypes) was assessed through the RXC test of independence with the log likelihood or G statistic (Sokal and Rohlf 1995), in which C (patients and controls) are one dimension of a tally of counts having R rows of distinct genetic categories (alleles, genotypes, or haplotypes). The test of significance has k-1 degrees of freedom. This procedure tests for differences between the overall patient and control distributions of a genetic category. If the distributions are different, then the individual contributions to that difference can be surveyed. Individual genetic category differences (say for an individual allele) are displayed with the OR and its confidence interval. ORs are converted to ln(OR)and then plotted to make positive and negative association space linearly equivalent, but the untransformed ORs are also given along the top of the figures. Following determination of nominally significant predisposing and protective alleles at HLA-A, -B, and DRB1 (with *P* set at <0.01), genotype and haplotype group categories are constructed from these deviant alleles to assess differences among these affective groupings: Favorable–protective (f) predisposing (p), and neutral (x) carrying chromosomes construct six possible genotypes (fx, ff, px, pp, fp, and xx). Similarly, the two locus haplotypes have a possible eight groups (fx, xf, ff, px, xp, pp, mixed [fp or pf] and xx). The ORs of the tallied groups are evaluated and compared.

The variation in disease susceptibility at the second allele in a genotype and at alleles at other loci on the same haplotype each modify the observed disease associated effects in another respect as well. Even a true allelic effect will, on average, be reduced by the larger HLA genetic context in which it occurs. This diminished measurement effect means that the true impact of alleles may be greater than that demonstrated.

One issue regarding attribution of the HLA disease effects deserves mention. If just one or a few alleles are protective of disease (e.g., the most common HLA-B alleles*07, *08, and *44, as observed here), then this combined effect could create the appearance of predisposing alleles at the other end of the predispositional spectrum even though these apparently susceptible alleles may be neutral with respect to disease. In this study, the extreme *p* values resulting from both the overall locus-wide tests and the individual *p* values of the predisposing alleles make such an explanation less likely.

### Declarations

The authors declare no conflicts of interest. The human data analyzed in this study were taken from existing databases containing no personal identifiers.

## Electronic Supplementary Material

Below is the link to the electronic supplementary material.

ESM 1 (PDF 421 kb)
