# Supplementary material for: Spectrum of HLA associations: the case of medically refractory pediatric acute lymphoblastic leukemia
Source: Immunogenetics. 2012 Feb 15;64(6):409–19. doi: 10.1007/s00251-012-0605-5 (PMC3349849; doi:10.1007/s00251-012-0605-5)
Supplement: Supplementary file 1 — (PDF 421 kb) [file 251_2012_605_MOESM1_ESM.pdf]

7/26/2011
Title: Spectrum of HLA Associations in Medically Refractory Pediatric Acute Lymphoblastic Leukemia

Journal: **Immunogenetics**

Corresponding Author: William Klitz, University of California, Berkeley, CA 94720-7360, USA, klitz@berekeley.edu

Supplemental Tables

Table S1. Case- Control Demographics

Sample Group (age yrs)	Total N	Female (%)	Male (%)	Geographic Region*
Cases (2-16)	2438	929 (38.1)	1509 (61.9)	Cases (1007 of 2438 with zip): East: 199 (19.8%), West:291 (28.9%), Midwest: 248 (24.6%), South: 269 (26.7%)
Controls (18-29)	41750	17133 (41.0)	24617 (59.0)	Controls (37616 of 41750 with zip): East: 7847 (20.9%) West: 10685 (28.4%) Midwest: 8793 (23.8%) South: 10291 (27.3%)

*Sample data was analyzed according to 4 geographical regions based on the first digit of the zip code (**0**,**1** = East; **2**,**3**,**7** = South; **4**,**5**,**6** = Midwest; **8**,**9** = West).

7/26/2011

Table S2. Class I HLA-A Alleles and pALL

Rank	Allele	pALL	freq	NCAU	freq	G	P	Odds	CI_Lower	CI_Upper	ln(Odds)	Ln(CI)	Ln(CI)
1	33	69	0.0142	1557	0.0187	5.3	0.0111651	0.761	0.598	0.968	-0.274	-0.514	-0.033
2	01	629	0.1290	13428	0.1608	36.5	7.962E-10	0.773	0.710	0.842	-0.257	-0.342	-0.172
3	03	592	0.1214	11836	0.1417	16.2	2.909E-05	0.837	0.767	0.914	-0.178	-0.265	-0.090
4	26	140	0.0287	2815	0.0337	3.6	0.0302953	0.850	0.716	1.009	-0.162	-0.333	0.009
5	66	21	0.0043	412	0.0049	0.3	0.3153445	0.892	0.579	1.374	-0.114	-0.547	0.318
6	25	89	0.0183	1697	0.0203	0.9	0.1722898	0.901	0.728	1.115	-0.104	-0.318	0.109
7	02	1359	0.2787	23573	0.2823	0.3	0.2998069	0.983	0.922	1.048	-0.018	-0.082	0.046
8	32	183	0.0375	2860	0.0343	1.5	0.1197402	1.102	0.947	1.282	0.097	-0.054	0.249
9	69	11	0.0023	178	0.0021	0.1	0.4718634	1.103	0.609	1.998	0.098	-0.496	0.692
10	11	325	0.0667	4990	0.0598	3.9	0.0277035	1.125	1.002	1.263	0.118	0.002	0.233
11	29	205	0.0420	3133	0.0375	2.6	0.0597795	1.128	0.977	1.302	0.121	-0.023	0.264
13	24	515	0.1056	7793	0.0933	8.0	0.0026135	1.148	1.045	1.261	0.138	0.044	0.232
14	31	160	0.0328	2157	0.0258	8.4	0.0023142	1.283	1.091	1.509	0.249	0.087	0.411
15	23	120	0.0246	1614	0.0193	6.4	0.0069876	1.285	1.066	1.548	0.251	0.064	0.437
16	Arare	5	0.0039	64	0.0334	0.4	0.4324	1.338	0.420	3.289	0.291	-0.867	1.191
17	30	155	0.0318	1917	0.0230	14.5	9.16E-05	1.401	1.188	1.653	0.337	0.172	0.503
18	68	275	0.0564	3298	0.0395	30.9	1.874E-08	1.456	1.284	1.651	0.376	0.250	0.501
19	34	9	0.0018	101	0.0012	1.7	0.1539816	1.604	0.827	3.110	0.472	-0.190	1.135
20	74	14	0.0029	76	0.0009	12.9	0.0004081	3.252	1.859	5.689	1.179	0.620	1.739
Total		4876	1.0000	83500	1.0000	145.3	1.80E-21						

7/26/2011

Table S3. Class I HLA-B Alleles and pALL

Rank	Allele	pALLcount	pcALL_freq	NCAU_count	NCAU_freq	G	P	Odds	CI_Lower	CI_Upper	ln(Odds)	Ln(CI)	Ln(CI)
1	14	131	0.0269	3862	0.0463	45.8	7.192E-12	0.571	0.479	0.681	-0.560	-0.735	-0.384
2	38	70	0.0144	1939	0.0232	18.1	1.124E-05	0.617	0.486	0.783	-0.483	-0.721	-0.245
3	08	379	0.0777	9118	0.1092	51.5	3.841E-13	0.688	0.619	0.766	-0.374	-0.480	-0.267
4	07	506	0.1038	10637	0.1274	24.4	4.216E-07	0.794	0.723	0.872	-0.231	-0.325	-0.137
5	57	151	0.0310	3131	0.0375	5.6	0.00927	0.823	0.698	0.970	-0.195	-0.360	-0.030
6	55	75	0.0154	1533	0.0184	2.2	0.0698912	0.840	0.667	1.060	-0.174	-0.405	0.058
7	44	636	0.1304	11902	0.1425	5.6	0.0092799	0.903	0.829	0.983	-0.102	-0.187	-0.017
8	35	496	0.1017	8116	0.0972	1.1	0.1560689	1.053	0.957	1.158	0.051	-0.044	0.146
9	27	203	0.0416	3294	0.0394	0.6	0.2335037	1.060	0.918	1.224	0.058	-0.086	0.202
10	52	59	0.0121	958	0.0115	0.2	0.3640244	1.064	0.818	1.382	0.062	-0.200	0.324
11	49	87	0.0178	1391	0.0167	0.4	0.2808752	1.078	0.868	1.339	0.075	-0.142	0.292
12	50	57	0.0117	892	0.0107	0.5	0.2724852	1.104	0.846	1.442	0.099	-0.168	0.366
13	56	32	0.0066	501	0.0060	0.3	0.3366875	1.110	0.779	1.582	0.105	-0.250	0.459
14	47	15	0.0031	232	0.0028	0.2	0.3889581	1.142	0.684	1.906	0.133	-0.380	0.645
15	15	393	0.0806	5729	0.0686	10.0	0.0009267	1.191	1.072	1.324	0.175	0.069	0.281
16	41	64	0.0131	914	0.0109	2.1	0.0919093	1.210	0.940	1.559	0.191	-0.062	0.444
17	37	81	0.0166	1154	0.0138	2.6	0.0631981	1.212	0.968	1.519	0.192	-0.033	0.418
18	45	36	0.0074	514	0.0062	1.2	0.1664021	1.216	0.870	1.701	0.196	-0.139	0.531
19	13	127	0.0261	1771	0.0212	5.0	0.0152913	1.239	1.033	1.484	0.214	0.033	0.395
20	58	51	0.0105	693	0.0083	2.6	0.0672269	1.274	0.960	1.691	0.242	-0.041	0.526
21	18	280	0.0574	3774	0.0452	14.9	7.011E-05	1.289	1.138	1.460	0.254	0.130	0.378
22	40	393	0.0806	5262	0.0630	22.4	1.377E-06	1.305	1.173	1.451	0.266	0.160	0.372
23	51	303	0.0621	4038	0.0484	17.6	1.652E-05	1.306	1.158	1.472	0.267	0.147	0.387
24	53	32	0.0066	312	0.0037	8.4	0.002965	1.786	1.245	2.561	0.580	0.219	0.940
25	39	175	0.0359	1574	0.0189	57.0	3.554E-14	1.942	1.659	2.275	0.664	0.506	0.822
26	*48*	*15*	*0.0031*	*104*	*0.0012*	*9.3*	*0.0023676*	*2.544*	*1.495*	*4.330*	*0.934*	*0.402*	*1.465*
27	*Brare*	*29*	*0.0059*	*156*	*0.0019*	*25.1*	*3.97E-07*	*3.197*	*2.071*	*4.780*	*1.162*	*0.728*	*1.564*
Total		*4876*	*1.0000*	*83500*	*1.0000*	*335.7*	*1.40E-55*						

7/26/2011

Table S4. HLA Class II DRB1 Alleles and pALL

Rank	Allele	pALLcount	pcALL_freq	NCAU_count	NCAU_freq	G	P	Odds	_CI_Lower	CI_Upper	ln(Odds)	Ln(CI)	Ln(CI)
1	01	454	0.0931	9823	0.1176	28.4	5.207E-08	0.771	0.699	0.850	-0.260	-0.359	-0.162
2	03	496	0.1017	9889	0.1184	12.8	0.0001847	0.844	0.767	0.927	-0.170	-0.265	-0.075
3	11	486	0.0997	9034	0.1082	3.5	0.0317818	0.913	0.830	1.005	-0.091	-0.186	0.005
4	07	601	0.1233	10615	0.1271	0.6	0.2222082	0.966	0.885	1.054	-0.035	-0.122	0.053
5	02	737	0.1511	12931	0.1549	0.5	0.249995	0.972	0.897	1.053	-0.028	-0.108	0.052
6	13	566	0.1161	9884	0.1184	0.2	0.324558	0.979	0.895	1.071	-0.021	-0.111	0.068
7	04	846	0.1735	13499	0.1617	4.7	0.015979	1.089	1.009	1.175	0.085	0.009	0.161
8	09	56	0.0115	829	0.0099	1.2	0.161482	1.168	0.892	1.530	0.155	-0.114	0.425
9	12	97	0.0199	1417	0.0170	2.4	0.0728274	1.181	0.961	1.452	0.167	-0.040	0.373
10	14	210	0.0431	2365	0.0283	31.6	1.35E-08	1.547	1.341	1.786	0.436	0.293	0.580
11	10	70	0.0144	761	0.0091	12.2	0.0003482	1.594	1.248	2.035	0.466	0.222	0.711
12	08	257	0.0527	2453	0.0294	71.1	2.683E-17	1.841	1.615	2.099	0.610	0.479	0.742
Total		4876	1.0000	83500	1.0000	159.4	1.80E-28						

7/26/2011

Table S5. Class I HLA-A Genotypic Classes and pALL

Genotype	# Genotypes	pALL Cases	Controls	OR	SE	CI Upper	CI Lower	ln(odds)	Ln(CI)	Ln(CI)
pp	10	164	1593	1.890436	0.085154	1.723534	2.057339	0.636808	0.544377	0.721413
px	31	559	8064	1.306583	0.050665	1.207278	1.405887	0.267415	0.188368	0.340668
ff	3	160	3951	0.694601	0.083844	0.530266	0.858936	-0.36442	-0.63438	-0.15206
fx	16	611	12327	0.829801	0.048969	0.733821	0.92578	-0.18657	-0.30949	-0.07712
fp	10	283	4913	1.023067	0.065553	0.894583	1.15155	0.022805	-0.1114	0.141109
xx	25	586	9862	1.071587	0.049746	0.974086	1.169088	0.069141	-0.02626	0.156224
totals	95	2363	40710							

7/26/2011

Table S6. Class I HLA-B Genotypic Classes and pALL

Genotype	# Genotypes	pALL Cases	Controls	OR	SE	CI Upper	CI Lower	ln(odds)	Ln(CI)	Ln(CI)
pp	14	260	2534	1.928257	0.069228	1.79257	2.063943	0.656616	0.58365	0.724618
px	33	365	4385	1.569947	0.059594	1.453142	1.686752	0.451042	0.373728	0.522805
ff	20	393	10020	0.626971	0.056944	0.515361	0.738581	-0.46686	-0.66289	-0.30302
fx	51	477	9704	0.83395	0.053154	0.729768	0.938131	-0.18158	-0.31503	-0.06387
fp	29	547	9739	0.992807	0.050816	0.893209	1.092406	-0.00722	-0.11294	0.088383
xx	15	127	2028	1.115749	0.094257	0.931006	1.300492	0.109526	-0.07149	0.262743
totals	162	2169	38410							

7/26/2011

Table S7. HLA Class II DRB1 Genotypic Classes and pALL

Genotype	# Genotypes	pALL Cases	Controls	OR	SE	CI Upper	CI Lower	ln(odds)	Ln(CI)	Ln(CI)
pp	6	205	2134	1.719056	0.076334	1.868670	1.56944	0.541775	0.625227	0.450720
px	18	664	10030	1.196761	0.047034	1.288949	1.104574	0.179619	0.253827	0.099460
ff	3	91	2380	0.646429	0.108936	0.859944	0.432915	-0.436291	-0.150888	-0.837215
fx	12	503	10484	0.782410	0.051400	0.883150	0.681670	-0.24538	-0.124260	-0.383209
fp	8	265	4466	1.026931	0.067024	1.158295	0.895566	0.026574	0.146949	-0.110300
xx	18	676	11991	0.962235	0.046640	1.053650	0.870821	-0.038496	0.052260	-0.138319
totals	65	2404	41485							

7/26/2011

Table S8. Class I HLA-A–HLA-B Haplotypic Classes and pALL

Haplotype	# Genotypes	pALL Cases	Controls	OR	SE	CI Upper	CI Lower	ln(odds)	Ln(CI)	Ln(CI)
ApBp	19	477.932	5195.293	1.63788	0.050249	1.736368	1.539391	0.493402	0.551795	0.431387
ApBx	20	299.8612	4814.192	1.071011	0.061431	1.191417	0.950606	0.068604	0.175144	-0.05066
AxBp	28	826.8634	11698.12	1.253404	0.039443	1.330713	1.176095	0.225863	0.285715	0.1622
AfBf	12	714.1129	16431.12	0.700375	0.041429	0.781576	0.619173	-0.35614	-0.24644	-0.47937
AfBx	20	287.0212	5393.394	0.905782	0.062452	1.028187	0.783377	-0.09896	0.027797	-0.24414
AxBf	30	807.3233	18011.47	0.721457	0.039436	0.798753	0.644162	-0.32648	-0.2247	-0.43981
mixed	31	506.9721	8588.722	1.012087	0.048282	1.10672	0.917454	0.012015	0.101401	-0.08615
AxBx	40	955.9139	13367.69	1.279337	0.037286	1.352418	1.206255	0.246342	0.301894	0.187521
totals	231	4876	83500							

7/26/2011

Table S9. HLA-B–DRB1 Haplotypic Classes and pALL

Haplotype	# Haplotypes	pALL Cases	Controls	OR	SE	Upper	Lower	ln(odds)	CI	CI
BpDRp	10	196.8408	1625.834	2.118449	0.07695	2.26927	1.967628	0.750684	0.819458	0.676829
BpDRx	33	1160.075	15905.15	1.326768	0.034768	1.394912	1.258623	0.282746	0.332832	0.230018
BxDRp	9	192.5108	1769.524	1.898511	0.077365	2.050147	1.746875	0.64107	0.717911	0.557828
BfDRf	11	476.582	11944.21	0.648978	0.049227	0.745462	0.552493	-0.43236	-0.29375	-0.59331
BfDRx	34	1308.865	26927.49	0.770877	0.033154	0.835858	0.705896	-0.26023	-0.1793	-0.34829
BxDRf	13	247.891	4567.671	0.925587	0.066946	1.0568	0.794374	-0.07733	0.055246	-0.2302
mixed	18	244.271	4144.68	1.00975	0.067554	1.142156	0.877343	0.009703	0.132918	-0.13086
BxDRx	52	1048.964	16615.44	1.103347	0.035913	1.173737	1.032957	0.098348	0.160193	0.032426
totals	180	4876	83500							
